# Ulcerated Scrotal Hemangioma in an 18-Month-Old Male Patient: A Case Report and Review of the Literature

**DOI:** 10.1155/2016/9236719

**Published:** 2016-06-16

**Authors:** Ioannis Patoulias, Konstantinos Farmakis, Christos Kaselas, Dimitrios Patoulias

**Affiliations:** 1st Pediatric Surgery Clinic of Aristotle University of Thessaloniki, G. H. G. Gennimatas, 41 Ethnikis Aminis Street, 54635 Thessaloniki, Greece

## Abstract

Deep scrotal hemangiomas are rare. Less than 50 case reports have been published. After systematic research of the literature, we found less than 5 cases of ulcerated scrotal hemangioma. The aim of this case report is to illustrate the challenges of scrotal hemangiomas pose and their potential therapies based on the successful surgical treatment of an ulcerated scrotal hemangioma in an 18-month-old male patient.

## 1. Introduction

Hemangiomas are the most common vascular tumors of childhood. Their incidence is historically reported as 1–3% of full term neonates and 10%–12% of children up to the age of 12 months [1, 2]. Recently, Munden et al. reported an incidence of 4.5% [3]. Hemangiomas can usually be found in head and neck, trunk, or extremities [3]. Hemangiomas localized at the scrotum are rare with less than 50 cases published [4–6]. Ulceration of the hemangioma is a known complication which occurs during its rapid growth phase and especially in anatomic areas where additional stimulating factors exist.

## 2. Aim

After analysis of the literature, we found less than 5 case reports of ulcerated scrotal hemangiomas. This observation was the motive for writing down the present study.

## 3. Case Report

An 18-month-old male patient was referred to our Outpatient Department seeking further consultation for a corrosive lesion over an already known hemangioma on the anterior surface of the scrotum. The patient was born at term without any abnormalities diagnosed during antenatal ultrasound scan and was free of any other medical history. The hemangioma was initially diagnosed 15 days after birth and its natural history of uncomplicated rapid growth was documented and followed by the family doctor. However, 40 days prior to consultation, a lesion at the hemangioma site was noted creating increased discomfort during urination and defecation of the patient.

Clinical examination confirmed the presence of a cavernous hemangioma measuring 2.5 × 2.3 cm, occupying almost the entire anterior surface of the scrotum. In addition, an extended ulceration covering almost the entire hemangioma surface (2.1 × 1.9 cm) was also observed.

Intrascrotal extension of the hemangioma was smooth at palpation, reaching deep in the scrotum, respectively, to the mesoscrotal diaphragm. No pathology was observed during the examination of intrascrotal structures of each hemiscrotum.

A color Doppler ultrasonographic evaluation of the scrotum, perineum, inguinal area, and pelvis confirmed the presence of increased vascular flow inside the mass and excluded its expansion to the lesser pelvis. Finally, the normal echo graphic morphology of the intrascrotal anatomic structures was demonstrated.

**Figure 1:**
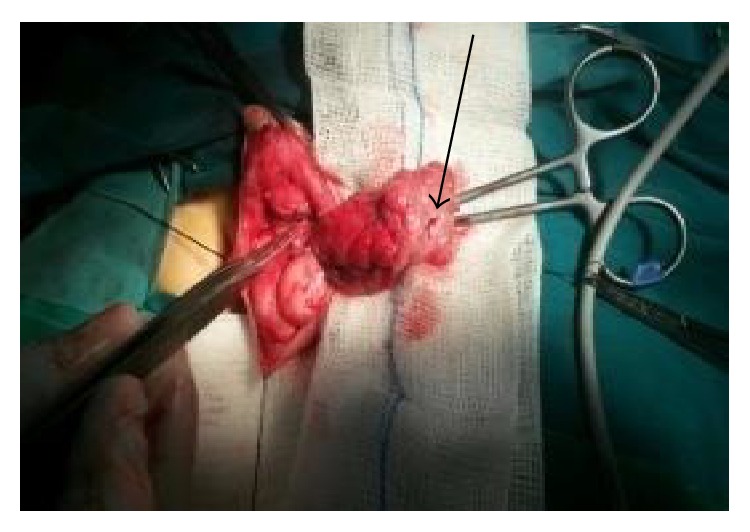
Progressive dissection with recognition and ligation with Vicryl 4-0 of the feeding vessels of the hemangioma. The ulceration on its scrotal part is observed (arrow).

**Figure 2:**
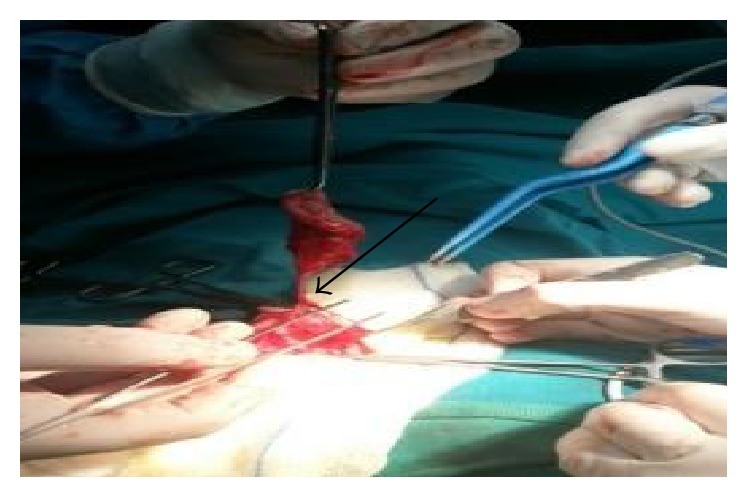
Demonstration of the main feeding vessel of the hemangioma, in the deepest part of the mesoscrotal diaphragm (arrow).

**Figure 3:**
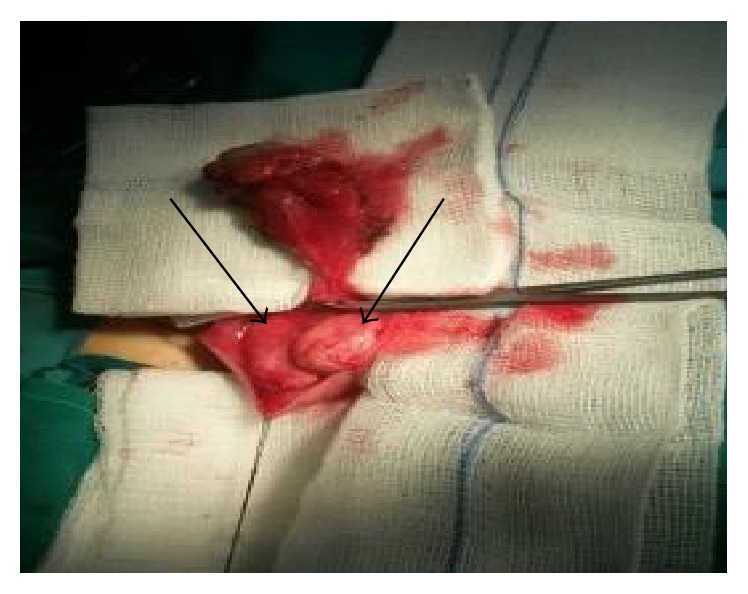
After the removal of the lesion, only the parietal layer of the tunica albuginea covers the testicles (arrows).

After discussion with the parents and following their preference, a decision for surgical excision was reached. Under general anesthesia, 4 stay sutures setting the skin limits of the lesion were placed. Following a tapered incision at the limits of the healthy scrotal skin and progressive dissection of the scrotal layers, the feeding vessels were identified and ligated (Figures 1, 2, and 3).

**Figure 4:**
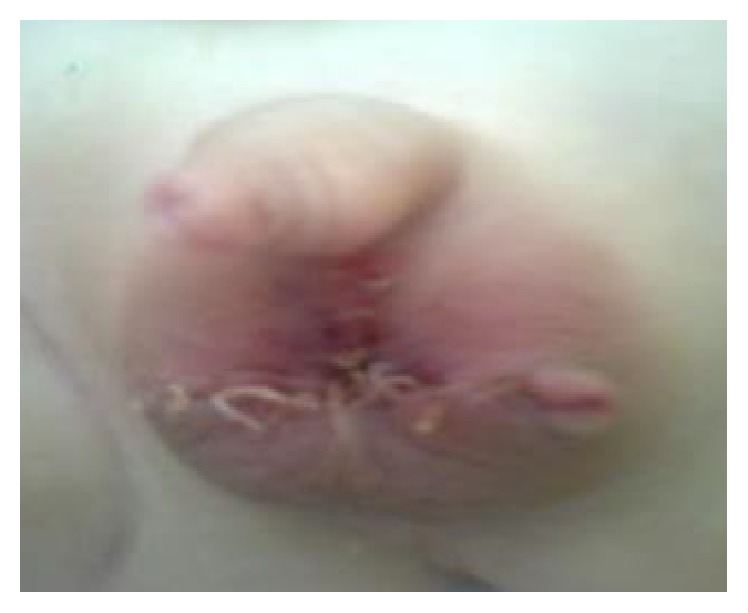
Postoperative view of the scrotum.

The significant scrotal defect that remained after excision of the hemangioma was restored by repositioning the ventricular part of the scrotum dorsally (Figure 4).

## 4. Results

**Figure 5:**
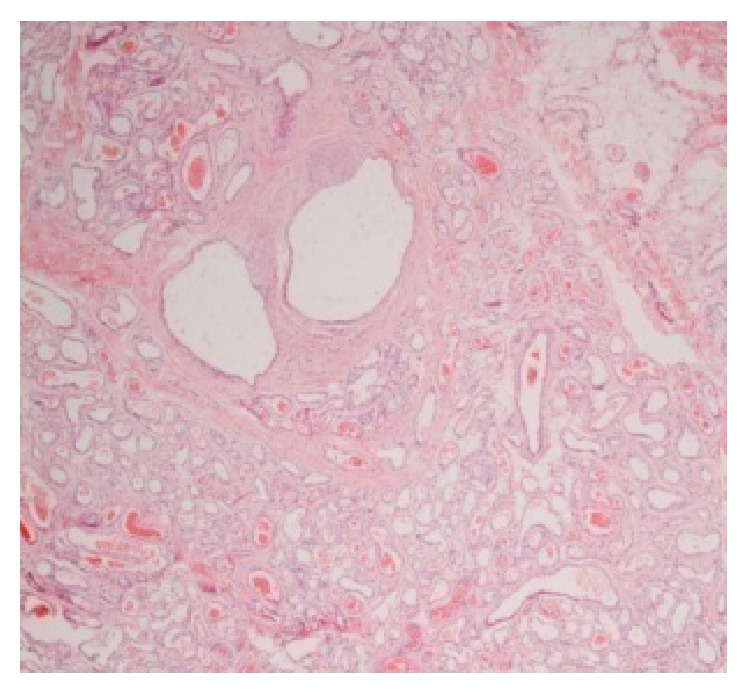
Presence of dilated tubular formations covered by an endothelial layer containing red blood cells, surrounded by thick fibrous connective tissue, was demonstrated (H-E, 40x).

The patient had an uncomplicated postoperative period and was discharged the second postoperative day. Histological examination confirmed the diagnosis of the cavernous hemangioma (Figure 5). At one-year follow-up, the patient remains asymptomatic without any recurrence of the lesion. Patient underwent ultrasound examination of the urinary tract and lesser pelvis, without pathology. The cosmetic result is satisfactory and intrascrotal structures are normal. Scrotal tissue has grown adequately to accommodate comfortably both testicles.

## 5. Discussion

Hemangiomas are the most common vascular lesions in childhood. Although scrotal hemangiomas are uncommon, they can create significant discomfort for both the patient and the family due to their location and potential complications. Complications that can be caused due to a deep scrotal hemangioma include hemorrhage, consequences of the extension of the lesion to the anatomical structures of the lesser pelvis (rectum, bladder) such as rectal bleeding and hematuria [7] ulceration with infection, hemorrhage due to trauma [8], and the potential effect on the spermatogenic activity of the testicles. The studies conducted by Stahl et al. and by Gotoh et al. [8, 9] demonstrated the harmful effect of the increased temperature of the hemangioma on the developing testicles.

The commonest complication of the lesion is ulceration which is believed to be triggered by exposure to stimulating factor such as the irritant action of secretions like urine and stools and the decreased oxygenation of the surface of the hemangioma [10]. Although the etiology of the development of such ulceration is unknown, the area covered by the diaper concerns 75% of hemangioma ulcerations (26% at buttocks, 23% at the perineum, 18% around the anal ring, and 5% at the scrotum) [10]. Another pathogenetic factor for the ulceration is considered to be the decreased oxygenation of the surface of the hemangioma [9]. Although uncommon, it is possible that, in the period of hemangiomas rapid growth, the offer/demand ratio of oxygenated blood is alternated. This conclusion is reinforced by the observation that ulceration is developed usually before the 10th month of life.

Although in our case the profound extension of the hemangioma was obvious, the development of a scrotal ulcer should be differentially diagnosed from trauma, herpes viral infection, intrauterine infection from varicella, infection from pyocyanin pseudomonas, aspergillus, pyogenic granuloma, pyoderma gangrenosum, and so forth [11]. If there is a profound lesion without superficial part, exclusion of the existence of a parascrotal tumor should be considered [12].

Imaging studies must determine the vascularization of the lesion, its relation with intrascrotal structures, and its potential expansion to the penis, perineum, and structures of the lesser pelvis [2, 13, 14]. We believe that MRI study should be performed when either due to the low vascular flow we cannot prove the existence of a hemangioma or an indirect presence of a hemangioma is revealed at the rectal or bladder wall (nodular thickening with echo graphic reflections) [2, 15]. Presence of hematuria could indicate an expansion to the bladder wall [16]. In our case, such a symptom was not noticed. As an alternative, we could perform a scintigraphy (Tc 99) to reveal a possible pelvic expansion of the lesion [15]. In our case, the vascular nature of the lesion was obvious by observation and increased blood flow was depicted at the color Doppler, without signs of expansion to the lateral structures, so we did not ask for further imaging studies (CT or MRI).

Oral propranolol is considered as the first-line treatment for infantile hemangiomas [17, 18]. The possible actions of propranolol include vasoconstriction, inhibition of angiogenesis, and induction of apoptosis. If treatment is given after the period of angiogenesis, propranolol is not beneficial for the patient. On the other hand, when treatment starts at the stage of proliferation, the growth of the lesion is inhibited. Thus, it is possible that when propranolol is ineffective, the proliferation stage has passed. Early diagnosis and treatment, ideally within the first six months of life, are crucial [17].

However, in cases of scrotal hemangiomas, many authors recommend the scheduled surgical removal of the lesion, with preservation of anatomic structures of the scrotum [5, 14, 16]. Enucleating of the lesion with minimal removal of testicular tissue is recommended only in the case of an endoscrotal hemangioma [19, 20]. In complicated superficial scrotal hemangiomas, a more conservative approach such as flash dye pulsed laser (CO_2_, Nd: YAG, yellow-light), infusion of NaCl 15%, and cryotherapy can be considered [2, 21–24]. Flash dye pulsed laser is a promising alternative to surgery that can achieve an selective photothermolysis and destruction of the superficial vessels of the lesion [24]. The completion of the treatment may demand 1–3 sessions. Healing of the ulcer occurs within the next following 2 weeks [25]. Sarig et al. describe in their study the successful treatment with the use of laser in 92, 8%, of all their patients, with a small complications rate (<3, 57%) and a satisfactory cosmetic result [26]. In our case, conservative and surgical treatment was discussed and offered to the parents. We, as the surgical team, were reluctant to administer propranolol, due to extension of ulceration and possible effects of increased temperature to future testicular function. As flash dye pulsed laser could not be offered in our hospital, a decision for surgical excision was reached.

In conclusion, both superficial and deep scrotal hemangiomas are rare lesions, which can induce increased discomfort to patients and their families due to their localization and their possible complications. It is crucial that conservative treatment with administration of propranolol starts within the stage of angiogenesis. However, in older babies or children, if no conservative treatment was initiated or has failed, laser treatment or surgical management and excision of the lesion should be considered.
